# First Report of the Biosynthesis and Characterization of Silver Nanoparticles Using *Scabiosa atropurpurea* subsp. *maritima* Fruit Extracts and Their Antioxidant, Antimicrobial and Cytotoxic Properties

**DOI:** 10.3390/nano12091585

**Published:** 2022-05-07

**Authors:** Badiaa Essghaier, Nourchéne Toukabri, Rihab Dridi, Hédia Hannachi, Inès Limam, Filomena Mottola, Mourad Mokni, Mohamed Faouzi Zid, Lucia Rocco, Mohamed Abdelkarim

**Affiliations:** 1 Department of Biology, Faculty of Sciences, University of Tunis El-Manar II, Tunis 2092, Tunisia; 2 Unité de Mycologie, Laboratoire de Recherche Infections et Santé Publique LR18SP01, Service de Dermatologie et de Vénéréologie, Hôpital La Rabta Jebbari, Tunis 1007, Tunisia; tnourchene@gmail.com (N.T.); mourad.mokni@rns.tn (M.M.); 3 Laboratoire de Matériaux, Cristallochimie et Thermodynamique Appliquée, Department of Chimie, Faculty of Sciences, University of Tunis El-Manar II, Tunis 2092, Tunisia; rihab018@live.fr (R.D.); medfaouzi.zid57@gmail.com (M.F.Z.); 4 Laboratory of Vegetable Productivity and Environmental Constraint LR18ES04, Department of Biology, Faculty of Science, University of Tunis El Manar II, Tunis 2092, Tunisia; hedia.hannachi@fst.utm.tn; 5 Laboratory of Oncohematology, PRF of Oncohematology, Faculty of Medicine of Tunis, Tunis El Manar University, Tunis 1006, Tunisia; limam.abdelkarim.ines@gmail.com (I.L.); mohamedabdelkarim2013@gmail.com (M.A.); 6 Department of Environmental, Biological and Pharmaceutical Sciences and Technologies (DiSTABiF), University of Campania “L. Vanvitelli”, 81100 Caserta, Italy; filomena.mottola@unicampania.it

**Keywords:** bio-synthetized silver nanoparticles, *Scabiosa atropurpurea* subsp. *maritima* (L.), antioxidants, antibacterial, anti-dermatophytes, cytotoxic activity

## Abstract

*Candida* and dermatophyte infections are difficult to treat due to increasing antifungal drugs resistance such as fluconazole, as well as the emergence of multi-resistance in clinical bacteria. Here, we first synthesized silver nanoparticles using aqueous fruit extracts from *Scabiosa atropurpurea* subsp. *maritima* (L.). The characterization of the AgNPs by means of UV, XRD, FTIR, and TEM showed that the AgNPs had a uniform spherical shape with average sizes of 40–50 nm. The biosynthesized AgNPs showed high antioxidant activity when investigated using 1,1-diphenyl-2-picryl-hydrazyl (DPPH) and ferric reducing antioxidant power (FRAP) assays. The AgNPs displayed strong antibacterial potential expressed by the maximum zone inhibition and the lowest MIC and MBC values. The AgNPs revealed a significant antifungal effect against the growth and biofilm of *Candida* species. In fact, the AgNPs were efficient against *Trichophyton rubrum, Trichophyton interdigitale,* and *Microsporum canis*. The antifungal mechanisms of action of the AgNPs seem to be due to the disruption of membrane integrity and a reduction in virulence factors (biofilm and hyphae formation and a reduction in germination). Finally, the silver nanoparticles also showed important cytotoxic activity against the human multiple myeloma U266 cell line and the human breast cancer cell line MDA-MB-231. Therefore, we describe new silver nanoparticles with promising biomedical application in the development of novel antimicrobial and anticancer agents.

## 1. Introduction

Metallic nanoparticles are thought to be the most promising anti-tumor and antimicrobial agents [1,2,3]. Green synthesis of nanoparticles based on the use of biological methods has increased due to their enhanced feasibility, biocompatibility, and increased environmental awareness [4,5]. The use of plant extracts for nanoparticle synthesis is more advantageous, providing a simple and rapid approach in comparison to the use of microorganisms, which requires aseptic and harsh microbial culture conditions [6,7]. Due to the richness of plant extracts with various compounds, research is ongoing in discovering new natural compounds with antimicrobial and antioxidant activities. Green synthesis routes for silver nanoparticles using plant species and various plants parts are emerging as promising antibacterial and anticancer agents owing various physicochemical properties and their safety for humans and the environment [8]. Several plant extracts have been reported, such as the flower extract of *Abelmpschus esculentus* (L) *Moench* [9]; the aqueous extract of pomegranate fruit peel (*Punica granatum*) [10]; the leaf extract of *Viburnum nervosum* [11]; methanolic, ethanolic, and aqueous extracts of *Achillea *millefolium** [12]; and *Picea abies* L. stem bark extract [13].

Moreover, extremophile plants have proven to be a source of various bioactive molecules owing to their antioxidant system adapted to environmental stress. Most species of *Scabiosa* belonging to the Caprifoliaceae family occur in the Mediterranean region [14,15]. In Tunisia, *Scabiosa* species were widely used as medicinal plants for skin treatment. Additionally, many works investigated *Scabiosa* species for their antioxidant and antibacterial properties, for example, *S*. *stellata* [16], *S*. *arenaria* [17], *S*. *atropurpurea*, *S*. *olivieri*, and *S*. *columbaria* [18,19]. However, *Scabiosa* species have not been widely studied in relation to cancer, except for *Scabiosa atropurpurea*, for which its anticancer activity against a lineage of colorectal cancer is known [20]. *Scabiosa* species are well known as high-antioxidant agents. Based on these data, we are pioneers in choosing *Scabiosa atropurpurea* subsp. *maritima* (L.) as reducing and stabilizing/capping agents required in the synthesis of natural silver nanoparticles [19,21]. To the best of our knowledge, there are no reports focusing on the biosynthesis of silver nanoparticles with *Scabiosa atropurpurea* subsp. *maritima* (L) extracts.

In recent years, a rapid increase in pathogen resistance to conventional antibiotics has been observed worldwide. Moreover, *Candida* infections present serious public health risks due to increased costs and hospitalization, as well as the increase in resistant fungal strains [22,23]. *Candida* species exposed to excessive doses of fluconazole acquire resistance by mutation [24,25]. Fungal infections have gained rising medical attention. Silver is known as a biocidal agent, by increasing permeability and cell death [26], and silver nanoparticles (AgNPs) are nontoxic to humans at very low concentrations, possessing high antimicrobial potential. AgNPs have revealed a significant role in various fields such as catalysis, pharmaceutical drug analysis, and biomedical applications [27]. They have also been extensively studied in the context of cancer treatment [3]. However, few studies on the antifungal effects of silver nanoparticles have been published, and little is known about their effects against pathogenic fungal causing skin infections. In this context, we should search for more efficient alternative antifungal and antibacterial agents.

The present work represents the first report on the synthesis of AgNPs using *Scabiosa atropurpurea* subsp. *maritima* (L) fruit extracts. The AgNPs were characterized by UV–Vis spectroscopy, Fourier transform infrared spectroscopy (FTIR), transmission electron microscopy (TEM), and X-ray diffraction (XRD). The antioxidant, antibacterial, antifungal, and antitoxic effects of the biosynthesized AgNPs were evaluated against clinical strains to examine their pharmaceutical and biomedical relevance.

## 2. Materials and Methods

### 2.1. Plant Collection and Extraction Procedure

The *Scabiosa atropurpurea* subsp. *maritima* fruits were collected from North Tunisia in July 2021. The plant material (20 mg) was washed with distilled water to remove debris and soil, dried at room temperature, and cut into small pieces. Then, it was added to 100 mL of distilled water and stirred at 50 °C for 30 min. Finally, the fruit extract was filtered with Whatman paper and stored at 4 °C until further use.

### 2.2. Synthesis of Nanoparticles

The biosynthesis of silver nanoparticles was conducted by the reaction mixture prepared by adding 7 mL of aqueous fruit extract and 5 mL of 5 mM AgNO_3_ solution, and then stirred for 4 min at room temperature. A visible color change was observed, from pale yellow to dark brown. Later, the synthesized silver nanoparticles (AgNPs) were separated and purified by continuous centrifugation (10,000 rpm; 15 min) with sterile milliQ water. The dried AgNPs were kept at 4 °C for further characterization [28].

### 2.3. Characterization of Synthesized Silver Nanoparticles

The formation of AgNPs was confirmed using several techniques in order to evaluate the functional aspects of the synthesized particles. The UV–Visible absorption spectrum was recorded using a 2802 UV/VIS spectrophotometer (UNICO). The shape and size of the AgNPs were determined by transmission electron microscope (TEM) [29] using FEI Tecnai F20 S/TEM. The X-ray diffraction (XRD) measurement was performed on an X-ray diffractometer (D8 ADVANCE BRUKER) using Cu K*_α_* radiation (*λ* = 1.5406 Å) [30]. The FTIR spectrum was recorded in the range 400–4000 cm^−1^ on a Varian FTIR 640 spectrophotometer with KBr pellets [31].

### 2.4. Clinical Microorganism Strain Origins and Culture Media

The antibacterial activity of AgNPs was investigated against clinical bacteria strains including *Klebsiella pneumoniae*, *Escherichia coli*, *Staphylococcus aureus*, and *Micrococcus luteus*. The reference strains of the *Candida* species (*Candida albicans*, *Candida tropicalis*, and *Candida glabrata*) and the dermatophytes species (*Trichophyton rubrum*, *Microsporum canis*, and *Trichophyton interdigitale*) were obtained by clinical isolation and identification using conventional methods at the dermatophytes laboratory services, La Rabta Hospital, Tunis, Tunisia. For the antibacterial and antifungal assays, Mueller–Hinton media (BioRad, France) and the Sabouraud chloramphenicol agar were used, respectively.

### 2.5. Antioxidant Activity

#### 2.5.1. Free Radical Scavenging Activity (DPPH)

The DPPH (2,2-Diphenyl-1-picryl-hydrazyl) free radical scavenging activity of the biosynthesized AgNPs was performed using the spectrophotometric method based on the reduction of DPPH (1, 1-diphenyl-2-picrylhydrazyl). The absorbance was measured at 517 nm, and the scavenging activity was calculated as percentage of inhibition (PI) at different sample concentrations (mg/mL), as recently published by Dridi and collaborators [28]. The assay was performed in triplicate.

#### 2.5.2. Ferric Antioxidant Reducing Power (FRAP)

The FRAP reagent containing 2.5 mL of a 10 mM TPTZ [2,4,6-tris(2pyridyl)-1,3,5-triazine] solution in 40 mM HCl, 2.5 mL of 20 mM ferric chloride, and 25 mL of 0.1 M acetate buffer (pH 3.6) was freshly prepared and warmed at 37 °C. The mixture reaction containing 100 µL of the AgNPs and 3 mL FRAP reagent was incubated at room temperature in the dark for 10 min. The absorbance was measured at 593 nm and converted by constructing a standard curve with ascorbic acid concentrations. The results were expressed as mg ascorbic acid equivalent antioxidant capacity (AEAC) per g of dry matter DM [32].

### 2.6. Antibacterial and Anti-Candida Assay by Agar Well Diffusion Method

The antibacterial and anti-*Candida* potentialities of AgNPs were investigated according to the method described by Dridi and collaborators [28]. The tests were performed in triplicate.

### 2.7. Effect on Cell Viability of Candida Species

A volume of 100 µL of individual *Candida* species suspensions (10^5^ spores/mL) were added to 1 mL of YM medium in the presence of 20 µL of the AgNPs (1 mg/mL). The incubation was performed at 37 °C on a shaker for about 48 h. The optical density was measured at 600 nm (A600). The growth inhibition was calculated as percent yeast survival = 100× (A600 of samples in the presence of AgNPs)/(A600 of negative control) as recently described by Dridi and collaborators [28].

### 2.8. Silver Nanoparticle Effects on Dermatophyte Growth, Mycelial Dry Weight, and Percentage Cellular Leakage

A volume of 100 µL of 10^5^ CFU/mL of *Trichophyton rubrum*, *Microsporum canis.* and *Trichophyton interdigitale* was added individually to 5 mL of Sabouraud dextrose broth (SDB) and 20 µL of AgNPs (1 mg/mL), incubated at 28 °C for 14 days in a rotary shaker to avoid precipitation of the AgNPs. The control tube consisted of an absence of AgNPs. Optical density was determined at 570 nm. The mycelial dry weight (in mg) was recuperated after centrifugation at 14,000 rpm for 20 min and dried at 60 °C [33]. The treated mycelia were incubated in saline solution for 30 min at 28 °C, after centrifugation at 12,000 rpm for 2 min, and the supernatants were used to determine the cellular leakage released, according to the work in [34]. The assay was performed in triplicate.

### 2.9. Biofilm Detection and Inhibition

Biofilm formation of the dermatophytes was performed by adding 200 µL of the dermatophytes (10^5^ spores/mL) to a 96-well plate; after 3 h of pre-adhesion, the wells were washed with 200 µL sterile saline to exclude non-adherent cells, and then 200 µL RPMI medium (Sigma-Aldrich, Taufkirchen, Germany) was added. The biofilm was detected at 630 nm according to the method by Brilhante and collaborators [35]. To evaluate the effect of AgNPs on biofilm formation of the dermatophytes, we added 10 µL of the silver nanoparticles at 1 mg/mL to the treated wells before use.

The possible effects of the AgNPs tested at 1 mg/mL on the biofilm formation of bacteria and *Candida* pathogen species was performed by using the crystal violet method as previously detailed [36,37].

### 2.10. Minimum Inhibitory Concentration (MIC), Minimum Bactericidal Concentration (MBC), and Minimum Fungicidal Concentration (MFC) Determinations

The MIC values were examined by the visible growth of microorganisms, using the standard broth dilution method in 96-well flat-bottomed microliter plates. The wells without visible microbial growth showed the MIC [38]. To determine the MBC and MFC, the method of Thakur et al. was applied [39]. The MBC/MIC and MFC/MIC ratios were calculated as described by Okou and collaborators [40] to examine the bactericidal and bacteriostatic effect, or the fungicidal and fungistatic effect.

### 2.11. Cell Culture

The human multiple myeloma U266 cell line was obtained from Leibniz Institute DSMZ (Braunschweig, Germany) and used as previously described [41,42]. The human breast cancer cell line MDA-MB-231 was kindly obtained from Dr. Khadija Essafi (Tunis Pasteur Institute, Tunis, Tunisia) [43]. Briefly, U266 and MDA-MB-231 cells were cultured in RPMI 1640 medium and Dulbecco’s minimal essential medium, respectively. Both of the media were supplemented with 10% heat-inactivated fetal bovine serum (FBS, PAN-Biotech, Aidenbach, Germany), 2 mM L-glutamine, and penicillin–streptomycin (PAN-Biotech, Aidenbach, Germany) at 37 °C and 5% CO_2_.

### 2.12. MTT Assay

The cytotoxic activities of the AgNPs were evaluated via an MTT assay. The MDA-MB-231 and U266 cells were seeded in 96-well culture plates for 24 h at 5 × 10^4^ and 2 × 10^5^ cells/mL/well, respectively. The MTT test was carried out after a period of treatment of 48 h with the AgNPs in concentrations ranging from 0.488 to31.125 μg/mL. In each experiment, the AgNPs were used as the vehicle control. The experiment was performed using three replicates for each culture condition and repeated three times [44].

### 2.13. Statistical Analysis

All the values are expressed as mean ± SEM. Comparisons between groups were performed using the generalized linear model (GLM) of the SAS statistical program. The multiple comparison of means was performed by using Student–Newman–Keuls SNK tests at a threshold of 5% (means with the same letters are not significantly different, n = 3).

## 3. Results

### 3.1. Characterization of Silver Nanoparticles

#### 3.1.1. Spectroscopic Analyses

**Figure 1:**
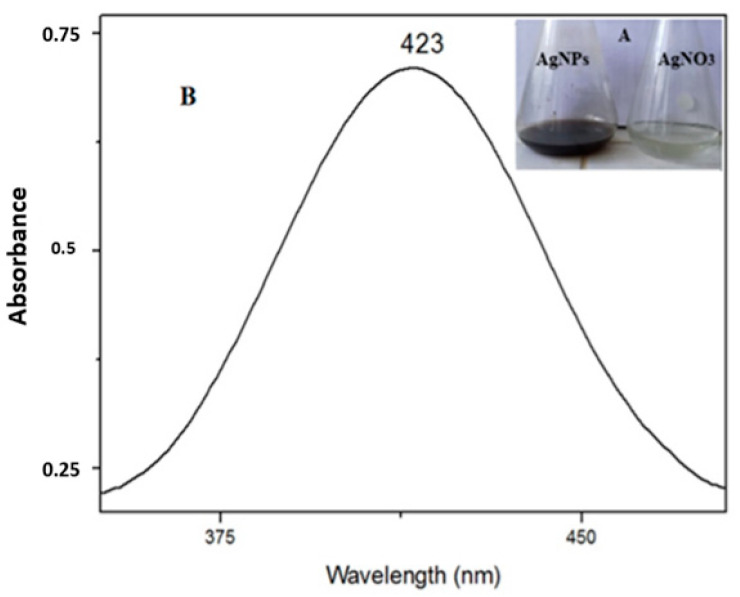
(**A**). Change in the color of the silver solution after adding plant extract, from light brown (right) to dark brown (left) and (**B**). UV–Vis spectrum of synthesized AgNPs (black) and the AgNO3 solution (before adding the plant extract (red)).

The UV–Vis spectroscopy demonstrated the bio-reduction of Ag nanoparticles from the aqueous AgNO_3_ solution. The solution containing silver nitrate turned dark brown after the addition of the studied fruit extracts, indicating the formation of AgNPs, while no color change was observed in the absence of plant extracts (Figure 1A). The characteristic surface plasmon resonance (SPR) absorption band of the biosynthesized AgNPs was obtained at 423 nm (Figure 1B).

FTIR measurements were used to identify the functional groups of the biomolecules present in the fruit extracts. These biomolecules are regarded to be the reducing agent for the silver ions and their interaction with the AgNPs. The FTIR technique used for the synthesized nanoparticles and plant extracts was examined (Figure S1). A consistent absorption peak found at 3397 cm^−1^ was referred to as the stretching of the OH group, and the broad spectrum at 2361 cm^−1^ corresponded to the C-H stretching vibration. The other observed bands around 1650, 1379, and 805 cm^−1^ were due to the stretching vibrations of the C=O, C-C, and C-N functional groups, respectively. The FTIR results indicate that the biological molecules present in *Scabiosa atropurpurea* subsp. *maritima* fruit extract could possibly be involved in both the synthesis and stabilization of AgNPs.

#### 3.1.2. Structural Study

**Figure 2:**
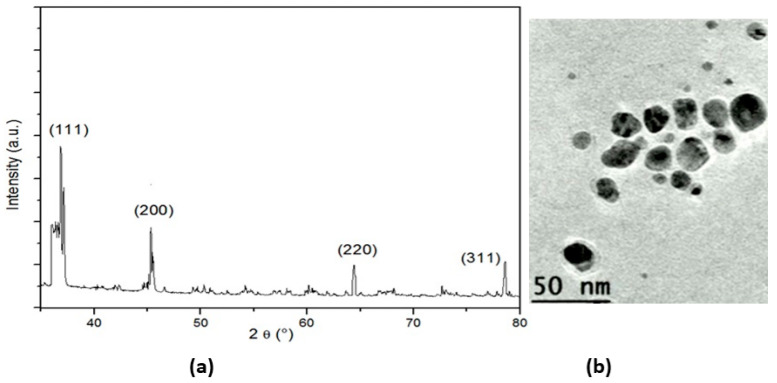
(**a**) XRD pattern of AgNPs; (**b**)TEM image of silver nanoparticles.

XRD was used for the phase identification and characterization of the nanoparticles’ crystal structure. The X-ray penetrated into the nanomaterial, and the resulting diffraction pattern was compared with the standards to obtain structural information (Figure 2a). The XRD analysis of the nanoparticles showed strong peaks corresponding to (111), (200) (220), and (311). The Bragg reflection was based on the face-centered cubic structure of the AgNPs. These planes confirmed the crystalline nature of the AgNPs. These peaks represented the 2θ° values of 37.51, 45.73, 66.43, and 77.38°, respectively. The nanoparticles showed a uniform spherical shape with an average size in the range of 40–50 nm (Figure 2b).

### 3.2. Antioxidant Activity of the Silver Nanoparticles

The DPPH free radical scavenging activity assay revealed that the AgNPs had antiradical activity. In addition, the IC50 value of the AgNPs was 0.112 mg/mL, comparable to the IC50 values of ascorbic acid with a value of 0.087 mg/mL. The synthesized silver nanoparticles were characterized by the high value of their ferric antioxidant reducing power of 0.036 mg EAa/g DM as compared to the ascorbic acid value of 0.024 (Table 1).

### 3.3. Antibacterial and Anti-Candida Activities

Overall, the results demonstrated the performance of the described AgNPs against bacteria and *Candida* clinical strains. *Escherchia coli* was the most sensitive bacteria strain, with a large zone inhibition (ZI) of 28 mm as compared to the other bacteria strains (ZI not exceeding 18 mm, as given by other published AgNPs). In addition, the described AgNP Sam was more active against the *Candida* species tested here, with higher ZI values as compared to other published AgNPs (Table 2).

**Figure 3:**
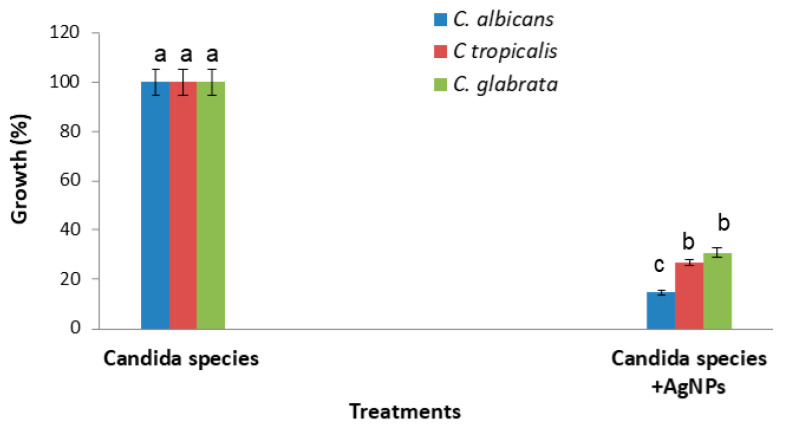
AgNPs’ effect on *Candida* growth after incubation for 48 h at 37 °C, as compared to untreated *Candida* cells. Error bars represent SE of the mean (n = 3). Means followed by the same letter are not significantly different according to the SNK test.

A strong inhibitory effect of the AgNPs on *Candida* growth was demonstrated against the *C. albicans* species, with an inhibition percentage of 85.26%, followed by 73.03% and 69.19% for *Candida tropicalis* and *Candida glabrata*, respectively (Figure 3).

The percentage of biofilm eradication was related to the microorganism’s strain; as a result, the AgNPs were more efficient on the biofilm of *Candida* with an inhibition percentage of more than 70.71% against the three tested *Candida* species. For the biofilm produced by bacteria, the results evidenced that an effective biofilm eradication was observed against *S*. *aureus*, with an inhibition percentage of 89.3%; for other bacteria, this percentage did not exceed 63.36%.

### 3.4. AgNPs Effect on Dermatophyte Growth, Mycelial Weight Dry, Cellular Leakage, and Biofilm Formation

The AgNPs markedly affected dermatophyte growth as determined by measuring the DO at 570 nm as well as the biomass dry weight of all the tested dermatophytes. A strong influence was noted on *Trichophyton rubrum* growth and on *Microsporum canis,* with fungalbiomass of 3 mg and 13.5 mg, respectively, as compared to their growth in the absence of AgNPs, where the growth was 44 mg and 30 mg, respectively. The biosynthesized AgNPs were also efficient in biofilm eradication of the dermatophyte species, with the percentage of biofilm eradication exceeding 82%. In addition, the presence of AgNPs increased the leaked materials of *Microsporum canis* by 68.3%, followed by 59.7% and 54.3% for *Trichophytom rubrum* and *Trichophytom interdigitale,* respectively. (Table 3).

**Figure 4:**
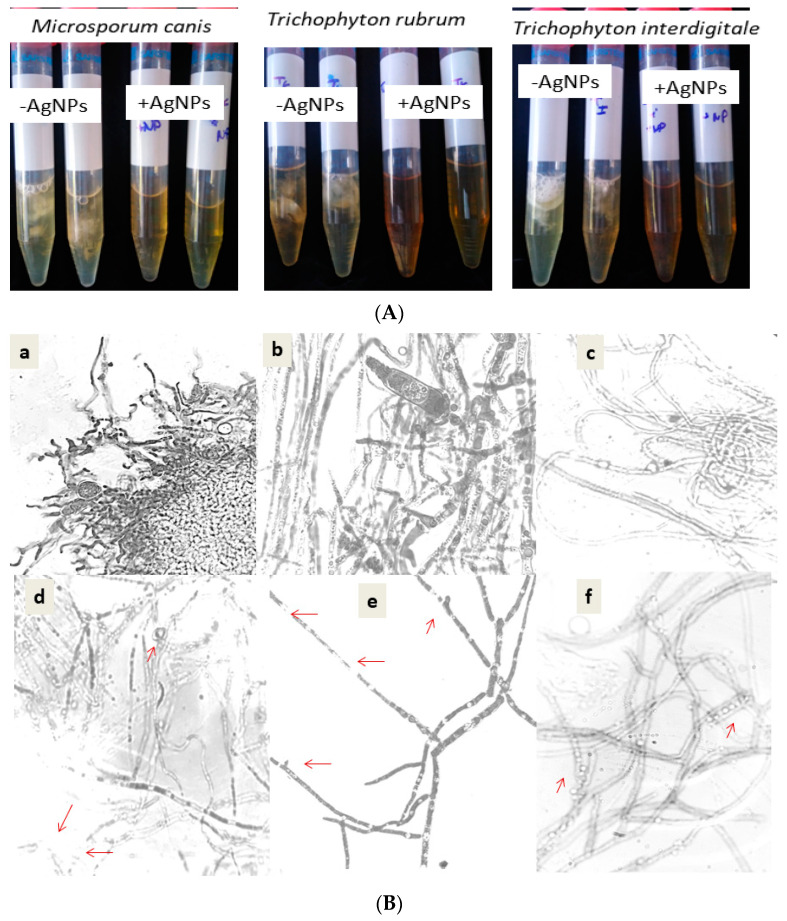
(**A**) Visual observation of dermatophyte growth after 14 days at 28 °C in the absence of AgNPs (AgNPs) and in the presence of AgNPs (+AgNPs). (**B**) Comparative effect of silver nanoparticles on the dermatophyte morphology of *Trichophyton interdigitale* (**d**), *Microsporum canis* (**e**), and *Trichophyton rubrum* (**f**), as compared to their control cultures (**a**–**c**), respectively. Red arrows indicate the morphological change by AgNPs.

Moreover, a visible effect on biofilm formation was indicated as showed by Figure 4A. During 14 days of exposure to the silver nanoparticles, no dermatophyte culture showed any hyphae or biofilm, which was conversely observed in the untreated culture with AgNPs (Figure 4B). These data were confirmed by the direct microscopic observation shown in Figure 4B. The microscopic observation of the dermatophyte culture proved the inhibitory effect on spore germination and hyphae ramification and alteration. The adherence of nanoparticles to the cell wall was clearly observed in the treated culture of dermatophytes. The change attacks the cell wall of the hyphae and decrease the ramification, with the absence of macroconidia. These changes were observed in the tested species (*Microsporum canis*, *Trichophyton rubrum*, and *Trichophyton interdigitale*).

### 3.5. MIC, MBC, and MFC Determinations

A markedly superior inhibitory effect was revealed against the dermatophyte species *Trichophyton rubrum* and *Trichophyton interdigitale*, with the lowest MIC values of 3.9 µg/mL. The silver nanoparticles exhibited a bactericidal and fungicidal effect as the MBC/MIC and MFC/MIC ratios did not exceed 4. The AgNPs effectively suppressed the growth of the tested pathogen due to the low values of the MIC, MBC, and MFC relative to those obtained by other published AgNPs. For example, the MIC and MBC values given by the described AgNPs were lower than those observed against the same bacterial strains, neither by the extract from *Scabiosa atrupurea*. Only the described silver nanoparticles showed a high antifungal action against the tested *Candida* and dermatophyte strains. (Table 4).

### 3.6. Cytotoxic Effect of AgNPs on the MDA-MB-231 and U266 Cancer Cell Lines

**Figure 5:**
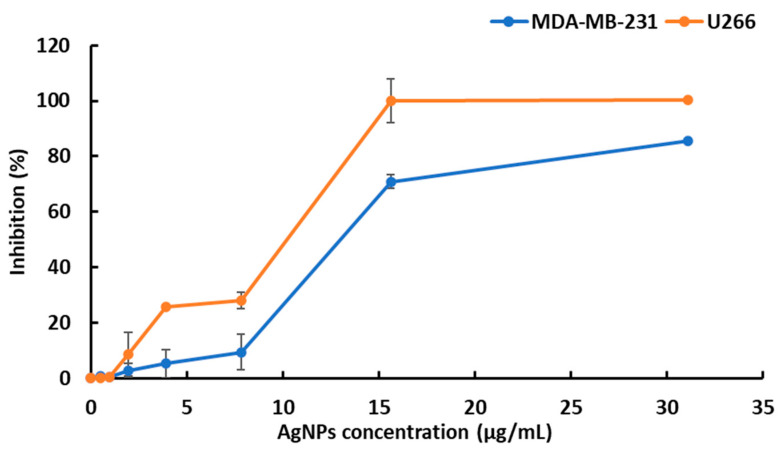
Evaluation of silver nanoparticle-induced cytotoxic effects on U266 and MDA-MB-231 cell lines. The reported values are the means ± SD from three independent experiments. No statistically significant differences between the control (no treatment) and the vehicle were noticed.

The biosynthesized silver nanoparticles showed cytotoxic activity on the MDA-MB-231 and U266 cell lines. The cytotoxic activity of the described AgNPs was assessed by an MTT assay on the metastatic breast cancer cell line MDA-MB-231 and the U266 cell lines. The two cell lines were responsive to AgNPs as observed on the viability curves (Figure 5). The results revealed that the silver nanoparticles significantly decreased cell viability in a dose-dependent manner. The U266 cells were more sensitive than MDA-MB-231 cells to AgNPs according to the calculated IC50 values at 48 h (10 and 12 μg/mL, respectively, for the U266 and MDA-MB-231 cell lines).

## 4. Discussion

The current study represents the first report on the biosynthesis of silver nanoparticles using *Scabiosa atropurpurea* subsp. *maritima* fruit and their biological potentialities. In the literature, it is well known that among the *Scabiosa* genus, the *Scabiosa atropurpurea* subsp. *maritima* species possesses the highest antioxidant activity, so we chose this plant as a reducing and stabilization/capping agent [19,21]. In fact, the *Scabiosa* species is an important source of phenolic compounds and flavonoids, which are the most important phytochemicals responsible for the antioxidant ability [17], related to the redox potential [49]. As regards its structure, the superior antioxidant potential given by the AgNPs may be correlated to the number of groups (OH) involved in the structure indicated by the FTIR analysis. In fact, phenolic groups facilitate the conversion of silver nitrate to AgNPs due to its electron-donating ability [50]. Recently, in their findings, the authors identified 19 phenolic compounds of *Scabiosa atropurpurea* subsp. *maritima* [48]. The biosynthesized AgNPs exhibited high antibacterial activity with low MIC and MBC values ranging from 7.81 to 31.25 µg/mL as compared to the data of published silver nanoparticles using other biological materials with MIC and MBC values ranging from 31 to 3500 µg/mL against bacteria. In the literature, antioxidant behavior is very important in biological applications such as in cancer therapy. Despite this, several studies revealed the high antioxidant activity of AgNPs from plant species [12,51,52]. The obtained DPPH value of 0.112 mg/mL of our AgNPs from the *Scabiosa* species proves their notable antioxidant properties as compared with the published findings of AgNPs from *Picea abies* L. with DPPH values ranging from 2.82 to 11.36 mg/mL [13], as well as the findings of AgNPs from various extracts of *Achillea *millefolium** exhibiting a greater inhibition of DPPH radicals with an IC50 value of 7.03 mg/mL [12]. In fact, it should be noted that genus *Scabiosa* has high antioxidant properties compared to other plants, which explains our choice of this plant species [48].

In addition, the results illustrate a strong antifungal activity, with the MIC and MFC values ranging from 3.9 to 62.5 µg/mL unlike other AgNPs with values from 62 to 1000 µg/mL, respectively [46,47,48]. For example, nanocides or nanotechnology-based pesticides, showed MIC values ranging from 200 to 180 µg/mL against *Microsporum canis* and *Trichophyton mentagophytes* [53]. In this research, the AgNPs showed high antifungal activity against *Candida* species, divergent from those reported for Se-NPs and Au-NPs [54]. This evidence highlights the efficacy of our studies on AgNPs against bacteria, *Candida*, and dermatophyte strains. Here, we propose their use to improve antifungal drugs. Considering the chemical characteristic of silver nanoparticles as having a metallic oxidative state and superficial area, the interaction of NPs with the surface of microorganisms increases. As a consequence, this property limits their growth. Based on the FTIR results, we suggest the incorporation of phenolic OH, amides, amines, and aldehyde in the AgNPs as stabilizing agents. Regarding the measured ZI, the described AgNPs had a greater ZI against the tested bacteria strains as opposed to other AgNPs (see Table 2). For example, the AgNPs from *Prosopis farcta* against the human pathogenic strains *S aureus* and *E coli* showed ZIs of 12 mm and 13.3 mm, respectively [50]. Moreover, the BOAgNPs synthesized by *Brassica oleracea* showed a weak ZI that did not exceed 10 mm against *Staphylococcus aureus*, *Klebsiella pneumoniae*, and *Escherchia coli* with an MIC of 25 µg/mL. These observations can prove the enhanced antibacterial and antifungal effect of our AgNPs from *Scabiosa atropurpurea* subsp *maritima.* Nanoparticles show many mechanisms of action. Among these, we cite cell wall damage, oxidative stress increases, and DNA interaction related to their nature, size, shape, and capping nature. The current work demonstrates the significant alterations to the morphogenesis of the fungal strains exposed to the synthesized AgNPs. Kim and collaborators [55] described the enhancement of the anti-dermatophytic activities of silver nanoparticles on *Candida* species and *Trichophyton mentagrophytes* as compared to the standard fluconazole. Moreover, the biosynthesized AgNPs generate organization changes and accumulation at the cell wall of the dermatophyte species *Trichophyton rubrum*, *Trichophyton interdigitale*, and *Microsporum canis*, as evidenced by the increase in the percentage of cell leakage. Similar observations were obtained for other silver nanoparticles able to disrupt the cell wall of the pathogen and thus inhibit its virulence to invasive tissue hosts [56]. In addition, we have recently reported the ability of silver nanoparticles from *Anagallis monelli* [28], as well as the use of a mixed-leaf extract of wild olive and pistachio [57] to prevent the dimorphic transition of *Candida albicans* from its yeast form to filamentous morphology and infection and penetration into human tissues. In dermatophytosis, the production of hyphae and biofilm is an important virulence factor in its ability to penetrate into the epidermis [58]. The accumulation of AgNPs on the cell wall and the hyphae of dermatophytes have been previously reported [59]. Similar effects were also observed for AgNPs on *Aspergillus brasiliensis* cells [60]. In the context of the current clinical results, the biocompatibility with skin cells and the outstanding antifungal performance of the biosynthesized nanoparticles might be exploited. Considering these discussions, here, we have described newly synthesized AgNPs that could be used as promising natural antifungal drugs able to prevent fungal pathogenesis. In the present work, the described silver nanoparticles demonstrated an inhibitory action against bacteria and *Candida* strains as compared to other reported silver nanoparticles, in addition to their superior antifungal action against dermatophytes. As regards the highest MICs values of 3.9 μg/mL given by our described AgNPs from *Scabiosa atropurpurea* subsp. *maritima* (L.), similar results have been observed for the nanoparticles reported by Mahmoudi et al. (2021) [61] in *T interdigitale* and *T. rubrum* giving MIC values of 3.73 μg/mL and 3.03 μg/mL, respectively, compared to an MIC of 4 μg/mL obtained with fluconazole. Moreover, recently there has been the detection of new dermatophyte strains resistant to current drugs such as terbinafine [62,63]. Faced with this situation, the described nanoparticles could be at least a potential alternative against cutaneous pathogens (dermatophytes and *Candida* species) resistant to conventional drugs, thus encouraging their industrial applications in skin care products and pharmacology associated with their exploitation in therapeutic and environmental applications.

The biosynthesized silver nanoparticles showed high cytotoxic and antiproliferative activities against the U266 and MDA-MB-231 cell lines in the MTT assays (IC50 values of 10 and 12 μg/mL, respectively). This finding proves the superior anticancer activity of our AgNPs compared to those described by Ansar et al. [64], who report a moderate effect of BOAgNPs on the cell viability of MCF-7 cell lines in an MTT assay (IC50 100 µg /mL). We also noticed that one report mentioned that *S*. *atropurourea* L. subsp. *maritima* (L.) enhanced doxorubicin cytotoxicity against Caco-2 colorectal cancer [20]. The significant cytotoxic activity of AgNPs could possibly be a result of the synergy between the bioactive compounds and the AgNPs and their ability to penetrate the cells, alter cell permeability, and cause cell death [60]. Recently, several pieces of research have focused on the synthesis of triangular silver nanoprisms (Ag-NPrs) due to their excessive biological properties, for example, their anisotropic shape, their plasmonic features in both visible and IR regions, and their significant SERS signals [65]. As an immediate objective of this work in the future, we suggest the conversion of our spherical nanoparticles to nanoprisms and a comparison of their biological potentialities.

## 5. Conclusions

This work is the first to deal with the simple method of biosynthesis of silver nanoparticles from *Scabiosa atropurpuerea subsp maritima* (L) and their remarkable antioxidant, antibacterial, and anti-*Candida* activities as compared to other silver nanoparticles. According to these results, our silver nanoparticles provide solid anti-dermatophytic properties affecting their virulence factor as a limitation of morphogenesis and cell leakage. In addition, the described AgNPs possess important anticancer activity. Based on these findings, the biosynthesized silver nanoparticles from *Scabiosa* could be used as promising antifungal drugs for long-term treatments, as it is preferable to select antifungal drugs with low MFCs and MICs that are able to prevent some pathological processes. In our future studies, we plan to apply methods such as the DCF assay and the COMET assay, which will allow us to better characterize the antioxidant and anti-genotoxic properties of our silver nanoparticles.

## Figures and Tables

**Table 1:** Antioxidant potential and antiradical capacity of AgNPs compared to ascorbic acid. DM: dry matter; EAa: equivalent ascorbic acid. Values are the average from triplicate experiments (mean n = 3). Different letters in the same column of each test show different significant differences at *p* < 0.05 using the SNK test.

**DPPH Free Radical Scavenging Activity IC50 (mg/mL)**	
AgNPs	0.112 ± 0.210 ^a^
ascorbic acid	0.087 ± 1.209 ^b^
**Ferric Antioxidant Reducing Power (FRAP) (mg EAa/gDM)**	
AgNPs	0.036 ± 0.225 ^a^
ascorbic acid	0.024 ± 0.101 ^b^

**Table 2:** Comparative zone inhibition (ZI) of clinical bacteria and *Candida* species, obtained by the described AgNPs of *Scabiosa atropurpurea* subsp. *maritima* (S. am) and other published AgNPs. Sam: aqueous extract of *Scabiosa atropurpurea* subsp. *maritima*; AWN: aqueous extract of *Phaenix dactylifer*; GWN: aqueous ethanolic extract of *Acacia milotica;* Sc: *Syzygium cumini*; Cg: supernatant of *Candida glabrata*.

Treatments	Antibacterial Activity ZI (mm)				Antifungal Activity ZI (mm)		
	*Staphylococcus aureus*	*Micrococcus luteus*	*Klebsiella pneumoniae*	*Escherchia coli*	*Candida albicans*	*Candida tropicalis*	*Candida glabrata*
AgNPs Sam	19.3 ± 0.57	26.3 ± 0.5	18.7 ± 0.28	28 ± 0.5	22 ± 0.28	24.7 ± 0.57	20.7 ± 0.5
AgNPsAWN [45]	17.8 ± 1.3			11.8 ± 1.0			
AgNPsGWN [45]	11.2 ± 0.1			12.0 ± 1.4			
AgNPs Sc [46]	20			18	22	17	nd
AgNPs Cg [47]	22		16	16	17	19	16

**Table 3:** Silver nanoparticles’ effect on dermatophyte growth, dry mycelial weight, cellular leakage, and biofilm inhibition. Values express the means of three repetitions.

Dermatophyte Strains	Cell Growth DO570 nm		Mycelial Dry Weight (mg)		Cellular Leakage (%)	Biofilm Inhibition BI (%)
	Untreated	AgNPs	Untreated	AgNPs	AgNPs	AgNPs
*T. rubrum*	0.376 ± 0.017	0.101 ± 0.09	44 ± 0.057	3 ± 0	59.7 ± 0.01	92 ± 0.102
*T. interdigitale*	0.331 ± 0.031	0.043 ± 0.015	59.6 ± 0.25	21.66 ± 0	54.3 ± 0.3	87 ± 0.05
*M.canis*	0.242 ± 0.067	0.102 ± 0.03	30 ± 0.01	13.5	68.3 ± 0.12	82 ± 0.02

**Table 4:** MIC, MBC, and MFC values of the silver nanoparticles against the clinical pathogenic strains as compared to other published silver nanoparticles. Values expressed in µg/mL are the average from triplicate experiments.

Bacterial Strains	AgNPs		AgNPs Sc [46]		AgNPs [47]		Scabiosa Atrupurea Extract [48]	
	MIC	MBC	MIC	MBC	MIC	MBC	MIC	MBC
*Escherchia coli*	15.62	31.25	125–250	250–500	31	62	1500	---
*Klebsiella pneumoniae*	15.62	15.62	---	---	62	125	---	---
*Staphylococcus aureus*	7.81	15.62	125–250	250–500	31	62	3500	3500
*Micrococcus luteus*	15.62	31.25	---	---	---	---	--	---
**Fungal Strains**	**MIC**	**MFC**	**MIC**	**MFC**	**MIC**	**MFC**	**MIC**	**MFC**
*Candida albicans*	7.81	31.25	125–250	250–500	62	125	1000	1000
*Candida tropicalis*	7.81	31.25	125–250	250–500	250	500	1000	1000
*Candida glabrata*	15.62	31.25	---	---	250	500	1000	1000
*Trichophyton ruburm*	3.9	62.5	---	---	---	---	---	---
*Trichophyton interdigitale*	3.9	62.5	---	---	---	---	---	---
*Microsporum canis*	15.62	62.5	---	---	---	---	---	---

## Data Availability

All data generated or analyzed during this study are included; any additional information is available from the corresponding author on reasonable request.

## Supplementary Materials

The following supporting information can be downloaded at: https://www.mdpi.com/article/10.3390/nano12091585/s1, Figure S1: Plant extract (Blue) and synthesized AgNPs (Black).
