# Supplementary material for: First Report of the Biosynthesis and Characterization of Silver Nanoparticles Using Scabiosa atropurpurea subsp. maritima Fruit Extracts and Their Antioxidant, Antimicrobial and Cytotoxic Properties
Source: Nanomaterials (Basel). 2022 May 7;12(9):1585. doi: 10.3390/nano12091585 (PMC9104986; doi:10.3390/nano12091585)
Supplement: Supplementary file 1 [file nanomaterials-12-01585-s001.zip › nanomaterials-1673249-supplementary.pdf]

First Report of the Biosynthesis and Characterization of Silver Nanoparticles Using *Scabiosa atropurpurea* subsp. *maritima* Fruit Extracts and Their Antioxidant, Antimicrobial and Cytotoxic Properties

Badiaa Essghaier [1,*], Nourchéne Toukabri [2], Rihab Dridi [3], Hédia Hannachi [4], Inès Limam [5], Filomena Mottola [6], Mourad Mokni [2], Mohamed Faouzi Zid [3], Lucia Rocco [6,*] and Mohamed Abdelkarim [5]

1 Department of Biology, Faculty of Sciences, University of Tunis El-Manar II, Tunis,2092 Tunisia
2 Unité de Mycologie, Laboratoire de Recherche Infections et Santé Publique LR18SP01, Service de Dermatologie et de Vénéréologie, Hôpital La Rabta Jebbari, Tunis 1007, Tunisia; tnourchene@gmail.com (N.T.); mourad.mokni@rns.tn (M.M.)
3 Laboratoire de Matériaux, Cristallochimie et Thermodynamique Appliquée, Department of Chimie,
Faculty of Sciences, University of Tunis El-Manar II, Tunis,2092 Tunisia; rihab018@live.fr (R.D.); medfaouzi.zid57@gmail.com (M.F.Z.)
4 Laboratory of Vegetable Productivity and Environmental Constraint LR18ES04, Department of Biology, Faculty of Science, University of Tunis El Manar II, Tunis,2092 Tunisia; hedia.hannachi@fst.utm.tn
5 Laboratory of Oncohematology, PRF of Oncohematology, Faculty of Medicine of Tunis, Tunis El Manar University, Tunis,1006 Tunisia; limam.abdelkarim.ines@gmail.com (I.L.); mohamedabdelkarim2013@gmail.com (M.A.)
6 Department of Environmental, Biological and Pharmaceutical Sciences and Technologies (DiSTABiF), University of Campania "L. Vanvitelli", Caserta, 81100 Italy; filomena.mottola@unicampania.it
*Correspondence: badiaaessghaier@gmail.com (B.E.); lucia.rocco@unicampania.it (L.R.)

Citation: Essghaier, B.; Toukabri, N.; Dridi, R.; Hannachi, H.; Limam, I.; Mottola, F.; Mokni, M.; Zid, M.F.; Rocco, L.; Abdelkarim, M. First Report of the Biosynthesis and Characterization of Silver Nanoparticles Using *Scabiosa atropurpurea* subsp. *maritima* Fruit Extracts and Their Antioxidant, Antimicrobial, and Cytotoxic Properties. *Nanomaterials* **2022**, *12*, x. https://doi.org/10.3390/xxxxx

Academic Editor(s): Heyou Han

Received: 31 March 2022
Accepted: 02 May 2022
Published: date

Publisher's Note: MDPI stays neutral with regard to jurisdictional claims in published maps and institutional affiliations.

Copyright: © 2022 by the authors. Submitted for possible open access publication under the terms and conditions of the Creative Commons Attribution (CC BY) license (https://creativecommons.org/licenses/by/4.0/).

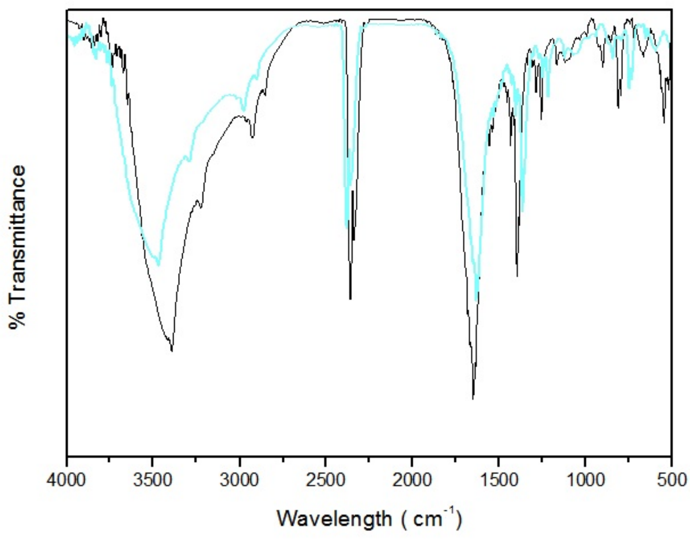

Figure S1. Plant extract (Blue) and synthesized AgNPs (Black)
